# Autoantibodies against type I IFNs in patients with critical influenza pneumonia

**DOI:** 10.1084/jem.20220514

**Published:** 2022-09-16

**Authors:** Qian Zhang, Andrés Pizzorno, Lisa Miorin, Paul Bastard, Adrian Gervais, Tom Le Voyer, Lucy Bizien, Jeremy Manry, Jérémie Rosain, Quentin Philippot, Kelian Goavec, Blandine Padey, Anastasija Cupic, Emilie Laurent, Kahina Saker, Martti Vanker, Karita Särekannu, COVID Human Genetic Effort, Laurent Abel, Alessandro Aiuti, Saleh Al-Muhsen, Fahd Al-Mulla, Mark S. Anderson, Evangelos Andreakos, Andrés A. Arias, Hagit Baris Feldman, Alexandre Belot, Catherine M. Biggs, Dusan Bogunovic, Alexandre Bolze, Anastasiia Bondarenko, Ahmed A. Bousfiha, Petter Brodin, Yenan Bryceson, Carlos D. Bustamante, Manish J. Butte, Giorgio Casari, John Christodoulou, Antonio Condino-Neto, Stefan N. Constantinescu, Megan A. Cooper, Clifton L. Dalgard, Murkesh Desai, Beth A. Drolet, Jamila El Baghdadi, Sara Espinosa-Padilla, Jacques Fellay, Carlos Flores, Paraskevi C. Fragkou, José Luis Franco, Antoine Froidure, Ioanna Evdokia Galani, Peter K. Gregersen, Bodo Grimbacher, Filomeen Haerynck, David Hagin, Rabih Halwani, Lennart Hammarström, James R. Heath, Sarah E. Henrickson, Elena W.Y. Hsieh, Eystein Husebye, Kohsuke Imai, Yuval Itan, Erich D. Jarvis, Timokratis Karamitros, Kai Kisand, Ourania Koltsida, Cheng-Lung Ku, Yu-Lung Lau, Yun Ling, Carrie L. Lucas, Tom Maniatis, Davood Mansouri, László Maródi, Isabelle Meyts, Joshua D. Milner, Kristina Mironska, Trine H. Mogensen, Tomohiro Morio, Lisa F.P. Ng, Luigi D. Notarangelo, Antonio Novelli, Giuseppe Novelli, Cliona O'Farrelly, Satoshi Okada, Keisuke Okamoto, Tayfun Ozcelik, Qiang Pan-Hammarström, Jean W. Pape, Rebeca Perez de Diego, David S. Perlin, Graziano Pesole, Anna M. Planas, Carolina Prando, Aurora Pujol, Lluis Quintana-Murci, Sathishkumar Ramaswamy, Vasiliki Rapti, Laurent Renia, Igor Resnick, Carlos Rodríguez-Gallego, Nikoletta Rovina, Vanessa Sancho-Shimizu, Anna Sediva, Mikko R.J. Seppänen, Mohammed Shahrooei, Anna Shcherbina, Ondrej Slaby, Andrew L. Snow, Pere Soler-Palacín, András N. Spaan, Ivan Tancevski, Stuart G. Tangye, Ahmad Abou Tayoun, Şehime Gülsün Temel, Sotirios Tsiodras, Stuart E. Turvey, K.M. Furkan Uddin, Mohammed J. Uddin, Diederik van de Beek, Donald C. Vinh, Horst von Bernuth, Joost Wauters, Mayana Zatz, Pawel Zawadzki, Helen C. Su, Jean-Laurent Casanova, Etablissement Français du Sang Study Group, Pascal Morel, Pascale Richard, Brigitte Bonneaudeau, Dorothée Cannet, Pierre Gallian, Michel Jeanne, Magali Perroquin, Hind Hamzeh-Cognasse, Fabrice Cognasse, Pierre Tiberghien, Constances Cohort, Rachel Nadif, Marcel Goldberg, Anna Ozguler, Joseph Henny, Sylvie Lemonnier, Mireille Coeuret-Pellicer, Stéphane Le Got, Marie Zins, 3C-Dijon Study, Christophe Tzourio, Stéphanie Debette, Carole Dufouil, Aïcha Soumaré, Morgane Lachaize, Nathalie Fievet, Amandine Flaig, Cerba HealthCare Group, Fernando Martin, Souad Mehlal-Sedkaoui, Jérôme Sallette, Lyon Antigrippe Working Group, Romain Hernu, Bruno Lina, Carole Schwebel, Isabelle Wroblewski, Patrice Morand, Bertrand Souweine, Benoit Boeuf, Helene Peigue-Lafeuille, Michael Darmon, Hugues Patural, Bruno Pozzetto, Jean Pierre Quenot, Benoit Colomb, Pierre Pothier, Alexandre Belot, REIPI INF Working Group, Maria Abad Arranz, Manuela Aguilar Guisado, Ana Escoresca Ortega, Rafaela Gallardo Ríos, Laura Merino Díaz, Maria Del Mar Muñoz Garcia, Nieves Ramírez Duque, Gloria María Romero Vázquez, Maria Jose Sánchez Cordero, Celia Salamanca Rivera, Jordi Niubó, Alexander Rombauts, Nicolás Navarrete, Laura Romero Oraa, Virginia Palomo, Tamara García-Salum, Marcela Ferres, Nicole Le Corre, Javier Sánchez-Céspedes, María Balsera-Manzanero, Jordi Carratala, Pilar Retamar-Gentil, Gabriela Abelenda-Alonso, Adoración Valiente, Pierre Tiberghien, Marie Zins, Stéphanie Debette, Isabelle Meyts, Filomeen Haerynck, Riccardo Castagnoli, Luigi D. Notarangelo, Luis I. Gonzalez-Granado, Nerea Dominguez-Pinilla, Evangelos Andreakos, Vasiliki Triantafyllia, Carlos Rodríguez-Gallego, Jordi Solé-Violán, José Juan Ruiz-Hernandez, Felipe Rodríguez de Castro, José Ferreres, Marisa Briones, Joost Wauters, Lore Vanderbeke, Simon Feys, Chen-Yen Kuo, Wei-Te Lei, Cheng-Lung Ku, Galit Tal, Amos Etzioni, Suhair Hanna, Thomas Fournet, Jean-Sebastien Casalegno, Gregory Queromes, Laurent Argaud, Etienne Javouhey, Manuel Rosa-Calatrava, Elisa Cordero, Teresa Aydillo, Rafael A. Medina, Kai Kisand, Anne Puel, Emmanuelle Jouanguy, Laurent Abel, Aurélie Cobat, Sophie Trouillet-Assant, Adolfo García-Sastre, Jean-Laurent Casanova

**Affiliations:** 1 St. Giles Laboratory of Human Genetics of Infectious Diseases, Rockefeller Branch, The Rockefeller University, New York, NY; 2 Laboratory of Human Genetics of Infectious Diseases, Necker Branch, INSERM U1163, Necker Hospital for Sick Children, Paris, France; 3 Université Paris Cité, Imagine Institute, Paris, France; 4 CIRI, Centre International de Recherche en Infectiologie - Team VirPath, Univ Lyon, INSERM U1111, Université Claude Bernard Lyon 1, CNRS UMR5308, ENS Lyon, Lyon, France; 5 Dept. of Microbiology, Icahn School of Medicine at Mount Sinai, New York, NY; 6 Global Health and Emerging Pathogens Institute, Icahn School of Medicine at Mount Sinai, New York, NY; 7 Dept. of Pediatrics, Necker Hospital for Sick Children, AP-HP, Paris, France; 8 Signia Therapeutics SAS, Lyon, France; 9 VirNext, Faculty of Medicine RTH Laennec, Claude Bernard Lyon 1 University, Lyon University, Lyon, France; 10 Joint Research Unit, Hospices Civils de Lyon-bioMérieux, Hospices Civils de Lyon, Lyon Sud Hospital, Pierre-Bénite, France; 11 Dept. of Pediatric Infectious Diseases and Immunology, School of Medicine, Pontificia Universidad Católica de Chile, Santiago, Chile; 12 Pathology Advanced Translational Research Unit, Dept. of Pathology and Laboratory Medicine, School of Medicine, Emory University, Atlanta, GA; 13 Center for Biomedical Research in Infectious Diseases Network (CIBERINFEC), Instituto de Salud Carlos III, Madrid, Spain; 14 Infectious Diseases, Microbiology and Preventive Medicine, Virgen del Rocío University Hospital, Sevilla, Spain; 15 Institute of Biomedicine of Seville (IBiS), CSIC, University of Seville, Seville, Spain; 16 Bellvitge Biomedical Research Institute (IDIBELL), Barcelona, Spain; 17 Dept. of Infectious Diseases, Bellvitge University Hospital, Barcelona, Spain; 18 University of Barcelona, Barcelona, Spain; 19 Infectious Diseases, Microbiology Unit, Virgen Macarena University Hospital, Seville, Spain; 20 Etablissement Francais Du Sang, La Plaine-Saint Denis, Saint-Denis, France; 21 University of Paris Cite, University of Paris-Saclay, UVSQ, INSERM UMS11, Villejuif, France; 22 University of Bordeaux, INSERM, Bordeaux Population Health Center, UMR1219, Bordeaux, France; 23 Laboratory for Inborn Errors of Immunity, Dept. of Microbiology, Immunology and Transplantation, KU Leuven, Leuven, Belgium; 24 Dept. of Pediatric Immunology and Pulmonology, Centre for Primary Immunodeficiency Ghent, PID Research Laboratory, Jeffrey Modell Diagnosis and Research Centre, Ghent University Hospital, Ghent, Belgium; 25 Laboratory of Clinical Immunology and Microbiology, Division of Intramural Research, National Institute of Allergy and Infectious Diseases, National Institutes of Health, Bethesda, MD; 26 Immunodeficiencies Unit, Hospital October 12, Research Institute Hospital October 12, School of Medicine, Complutense University, Madrid, Spain; 27 Pediatrics Service, Hematology and Oncology Unit, University Hospital 12 October, Madrid, Spain; 28 Laboratory of Immunobiology, Center for Clinical, Experimental Surgery and Translational Research, Biomedical Research Foundation of the Academy of Athens, Athens, Greece; 29 Dept. of Immunology, University Hospital of Gran Canaria Dr. Negrín, Canarian Health System, Las Palmas de Gran Canaria, Spain; 30 Dept. of Clinical Sciences, University Fernando Pessoa Canarias, Las Palmas de Gran Canaria, Spain; 31 Critical Care Unit, University Hospital of Gran Canaria Dr. Negrin, Canarian Health System, Las Palmas de Gran Canaria, Spain; 32 CIBER de Enfermedades Respiratorias (CIBERES), Instituto de Salud Carlos III, Madrid, Spain; 33 Dept. of Internal Medicine, University Hospital of Gran Canaria Dr. Negrin, Canarian Health System, Las Palmas de Gran Canaria, Spain; 34 Dept. of Respiratory Diseases, University Hospital of Gran Canaria Dr. Negrin, Canarian Health System, Las Palmas de Gran Canaria, Spain; 35 Dept. of Medical and Surgical Sciences, School of Medicine, Universidad de Las Palmas de Gran Canaria, Las Palmas de Gran Canaria, Spain; 36 Critical Care Unit, Hospital Clínico de Valencia, Valencia, Spain; 37 INCLIVA Biomedical Research Institute, Valencia, Spain; 38 Dept. of Respiratory Diseases, Hospital Clínico y Universitario de Valencia, Valencia, Spain; 39 Dept. of General Internal Medicine, Medical Intensive Care Unit, University Hospitals Leuven, Leuven, Belgium; 40 Laboratory of Human Immunology and Infectious Disease, Graduate Institute of Clinical Medical Sciences, Chang Gung University, Taoyuan, Taiwan; 41 Division of Infectious Diseases, Dept. of Pediatrics, Chang Gung Memorial Hospital, Taoyuan, Taiwan; 42 Dept. of Pediatrics, Hsinchu MacKay Memorial Hospital, Hsinchu, Taiwan; 43 Dept. of Nephrology, Chang Gung Memorial Hospital, Taoyuan, Taiwan; 44 Center for Molecular and Clinical Immunology, Chang Gung University, Taoyuan, Taiwan; 45 Metabolic Clinic, Ruth Rappaport Children's Hospital, Rambam Health Care Campus, Haifa, Israel; 46 Rappaport Faculty of Medicine, Technion Institute of Technology, Haifa, Israel; 47 Etablissement Français Du Sang, Université de Franche-Comté, Besançon, France; 48 Virology Laboratory, CNR des Virus des Infections Respiratoires, Institut des Agents Infectieux, Hôpital de la Croix Rousse, Hospices Civils de Lyon, Lyon, France; 49 Medical Intensive Care Dept., Hospices Civils de Lyon, Edouard Herriot Hospital, Lyon, France; 50 Pediatric Intensive Care Unit, Hospices Civils de Lyon, Hopital Femme Mère Enfant, Lyon, France; 51 Dept. of Medicine, School of Medicine, University of Seville, Seville, Spain; 52 Institute of Biomedicine and Translational Medicine, University of Tartu, Tartu, Estonia; 53 Dept. of Medicine, Division of Infectious Diseases, Icahn School of Medicine at Mount Sinai, New York, NY; 54 The Tisch Cancer Institute, Icahn School of Medicine at Mount Sinai, New York, NY; 55 Dept. of Pathology, Molecular and Cell-Based Medicine, Icahn School of Medicine at Mount Sinai, New York, NY; 56 Howard Hughes Medical Institute, New York, NY

## Abstract

Autoantibodies neutralizing type I interferons (IFNs) can underlie critical COVID-19 pneumonia and yellow fever vaccine disease. We report here on 13 patients harboring autoantibodies neutralizing IFN-α2 alone (five patients) or with IFN-ω (eight patients) from a cohort of 279 patients (4.7%) aged 6–73 yr with critical influenza pneumonia. Nine and four patients had antibodies neutralizing high and low concentrations, respectively, of IFN-α2, and six and two patients had antibodies neutralizing high and low concentrations, respectively, of IFN-ω. The patients’ autoantibodies increased influenza A virus replication in both A549 cells and reconstituted human airway epithelia. The prevalence of these antibodies was significantly higher than that in the general population for patients <70 yr of age (5.7 vs. 1.1%, P = 2.2 × 10^−5^), but not >70 yr of age (3.1 vs. 4.4%, P = 0.68). The risk of critical influenza was highest in patients with antibodies neutralizing high concentrations of both IFN-α2 and IFN-ω (OR = 11.7, P = 1.3 × 10^−5^), especially those <70 yr old (OR = 139.9, P = 3.1 × 10^−10^). We also identified 10 patients in additional influenza patient cohorts. Autoantibodies neutralizing type I IFNs account for ∼5% of cases of life-threatening influenza pneumonia in patients <70 yr old.

## Teaser

In an international cohort of 279 patients with hypoxemic influenza pneumonia, we identified 13 patients (4.6%) with autoantibodies neutralizing IFN-α and/or -ω, which were previously reported to underlie 15% cases of life-threatening COVID-19 pneumonia and one third of severe adverse reactions to live-attenuated yellow fever vaccine.

## Graphical Abstract

**Figure:**
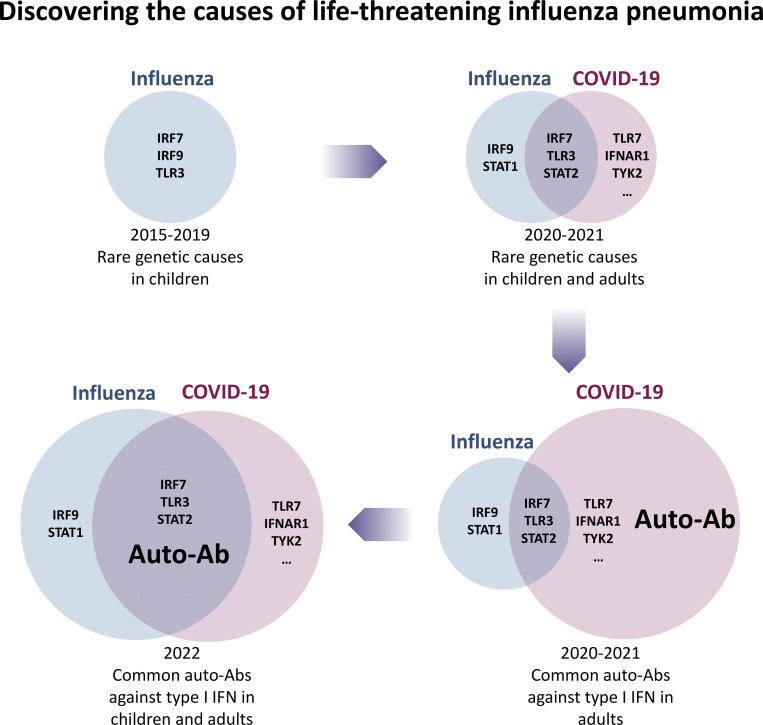

## Introduction

Seasonal influenza viruses (influenza A and B viruses [IAV and IBV]) infect ∼18% of unvaccinated people each winter, ∼17% of whom require medical attention (Hayward et al., 2014). Most unvaccinated infected individuals present with only asymptomatic infection or self-limited disease, but seasonal influenza nevertheless accounts for ∼400,000 deaths from respiratory causes per year worldwide (Iuliano et al., 2018; Paget et al., 2019; Krammer et al., 2018) and ∼10% of admissions and deaths from respiratory disease in hospitals (Cromer et al., 2014). Mortality rates are even higher for virulent, pandemic influenza viruses (Krammer et al., 2018). Why do a minority of infected individuals suffer from life-threatening seasonal influenza, whereas the majority do not? Different viral strains can explain year-to-year or region-to-region differences in mortality (Medina and Garcia-Sastre, 2011; Tscherne and Garcia-Sastre, 2011), but not interindividual variability within a given region and time period (Casanova and Abel, 2020, 2021a, 2021b, 2022; Zhang et al., 2022). Efforts have been made to identify human epidemiological risk factors. Aging is the major epidemiological determinant of death from influenza infection (Cromer et al., 2014; Paget et al., 2019; Iuliano et al., 2018; Krammer et al., 2018). Despite differences in overall mortality between the 32 countries with various levels of vaccination coverage studied, it is clear that people aged 65–75 yr, and those >75 yr old, are 7–38 and 24–249 times more likely, respectively, to die from respiratory influenza infection than people <65 yr old (Iuliano et al., 2018). The age-dependent increase in the risk of death, and the sharp increase in people >65 yr old in particular, remain unexplained, but has also been reported for other respiratory viruses, including adenovirus, respiratory syncytial virus (Watson and Wilkinson, 2021), and SARS-CoV-2 (Piroth et al., 2020), suggesting the possibility of shared immunological mechanisms. A few comorbid conditions, such as chronic pulmonary diseases, are associated with life-threatening influenza (Cromer et al., 2014). Both anti-influenza vaccination and infections with influenza viruses confer some protection against specific and, to a lesser extent, cross-reactive influenza viruses (Krammer et al., 2018; Kostova et al., 2013). However, a lack of immune memory is not, in itself, sufficient to cause critical influenza, as demonstrated by patients with inherited and acquired deficiencies of T and B cell adaptive immunity, whose impaired antibody responses to the influenza vaccine do not seem to create a predisposition to critical influenza (Zhang, 2020). Most, if not all, life-threatening cases of influenza in vaccinated and unvaccinated individuals, including those >65 yr old, remain unexplained at the molecular and cellular levels.

A first breakthrough came from human genetic studies of rare children with life-threatening influenza pneumonia. In 2015, we reported autosomal recessive (AR) IRF7 deficiency in an otherwise healthy 7-yr-old girl who had suffered from life-threatening influenza pneumonia at the age of 3 yr (Ciancanelli et al., 2015). She has since remained well with only annual influenza vaccinations and vaccination against COVID-19 for prophylaxis. Two other patients with IRF7 deficiency suffering from severe influenza pneumonia at the ages of 7 mo and 14 yr have recently been reported (Campbell et al., 2022). IRF7 is a transcription factor required for the production of the 17 type I IFNs and three type III IFNs, with IFN-β not being strictly IRF7 dependent in some cell types (Ciancanelli et al., 2015; Zhang et al., 2020b; Campbell et al., 2022). Plasmacytoid dendritic cells from these patients produced no type I and III IFNs other than IFN-β in response to IAV (Ciancanelli et al., 2015; Campbell et al., 2022). Moreover, a 2-yr-old child with AR IRF9 deficiency (Hernandez et al., 2018); three children with autosomal dominant TLR3 deficiency (Lim et al., 2019), aged 5 wk and 5 and 9 yr; three children with AR STAT1 deficiency, including two aged 1 mo and one aged 6 mo (Le Voyer et al., 2021); and a 10-mo-old child with AR STAT2 deficiency (Freij et al., 2020) have all been reported to have suffered from life-threatening influenza pneumonia. TLR3 is an endosomal sensor of dsRNA that controls tonic type I IFN levels in at least some nonhematopoietic cells (Gao et al., 2021), whereas STAT1, STAT2, and IRF9 are the three components of the type I and III IFN-driven ISGF3 transcription factor (Zhang, 2020). Both IRF7- and TLR3-deficient respiratory epithelial cells (RECs) derived from patients’ induced pluripotent stem cells fail to control IAV replication (Lim et al., 2019; Ciancanelli et al., 2015), a phenotype rescued by exogenous type I or III IFN. These five genetic etiologies of life-threatening influenza pneumonia thus impair type I and III IFN immunity to IAV. These cases revealed the indispensable role of human intrinsic (TLR3, IRF7, IRF9, STAT1, and STAT2 in RECs, in which the virus replicates) and innate (IRF7 in plasmacytoid dendritic cells, in which the virus does not replicate) type I and III IFN immunity in host defense against influenza (Casanova and Abel, 2021b, 2022; Duncan et al., 2021; Manry et al., 2022; Zhang et al., 2022).

The genetic study of critical influenza pneumonia led to that of critical COVID-19 pneumonia (Casanova and Abel, 2021b, 2022; Zhang et al., 2022). The COVID Human Genetic Effort (http://www.covidhge.com; Casanova et al., 2020) found inborn errors of TLR3-dependent or -independent type I IFN immunity, including not only AR IRF7 deficiency but also AR IFNAR1 deficiency, in previously healthy patients with critical COVID-19 (Zhang et al., 2020b, 2022, Casanova and Abel, 2021b, 2022; Abolhassani et al., 2022; Campbell et al., 2022). Following on from the 1984 description of autoantibodies (auto-Abs) against type I IFNs in a single patient with disseminated zoster (Pozzetto et al., 1984), we showed that preexisting auto-Abs neutralizing type I IFNs underlie ≥15% of cases of life-threatening COVID-19 pneumonia (Bastard et al., 2020; Bastard et al., 2021a; Zhang et al., 2022; Puel et al., 2022) and 30% of severe adverse reactions to the yellow fever vaccine (Bastard et al., 2021c). These findings have since been widely replicated (Abers et al., 2021; Acosta-Ampudia et al., 2021; Bastard et al., 2021d; Chang et al., 2021; Chauvineau-Grenier et al., 2021; Goncalves et al., 2021; Koning et al., 2021; Lemarquis et al., 2021; Meisel et al., 2021; Savvateeva et al., 2021; Solanich et al., 2021; Troya et al., 2021; Van Der Wijst et al., 2021; Vazquez et al., 2021; Wang et al., 2021; Ziegler et al., 2021; Akbil et al., 2022; Busnadiego et al., 2022; Carapito et al., 2022; Credle et al., 2022; Eto et al., 2022; Frasca et al., 2022; Lamacchia et al., 2022; Mathian et al., 2022; Raadsen et al., 2022; Simula et al., 2022; Soltani-Zangbar et al., 2022). Individuals with auto-Abs against type I IFNs are, thus, susceptible to at least two life-threatening viral infections. These auto-Abs can be genetically driven, as in patients with autoimmune polyendocrinopathy syndrome type 1 (APS-1) due to *AIRE* mutations (Bastard et al., 2021d), T cell deficits due to hypomorphic *RAG1* or *RAG2* mutations (Walter et al., 2015), immune dysregulation, polyendocrinopathy, enteropathy, X-linked due to *FOXP3* mutations (Rosenberg et al., 2018), or incontinentia pigmenti due to *NEMO* mutations (Harris et al., 1992; Bastard et al., 2020). These auto-Abs are also found in patients treated with IFN-α or IFN-β (Vallbracht et al., 1981; Rudick et al., 1998) or with systemic lupus erythematosus (Panem et al., 1982; Gupta et al., 2016), thymoma (Shiono et al., 2003), or myasthenia gravis (Bello-Rivero et al., 2004; Meager et al., 2003). Plasma containing such auto-Abs (diluted 1:10) can neutralize low (100 pg/ml) or high (10 ng/ml) concentrations of the 13 types of IFN-α and/or IFN-ω. The neutralization of IFN-β (10 ng/ml) is rarer. Remarkably, we showed that these auto-Abs are common in the general population, being present in 1% of individuals <70 yr old, 2.3% of those 70–80 yr old, and 6.3% of those >80 yr old (Bastard et al., 2021a). These auto-Abs are the second most common determinant of COVID-19 death after age (Zhang et al., 2020a, 2022; Bastard et al., 2021a; Casanova and Abel, 2021b, 2022; Manry et al., 2022; Puel et al., 2022). We therefore hypothesized that auto-Abs neutralizing type I IFNs might also underlie life-threatening influenza pneumonia.

## Results

### Auto-Abs neutralizing IFN-α2 in 13 of 279 patients (4.7%) with critical influenza

**Figure 1.**
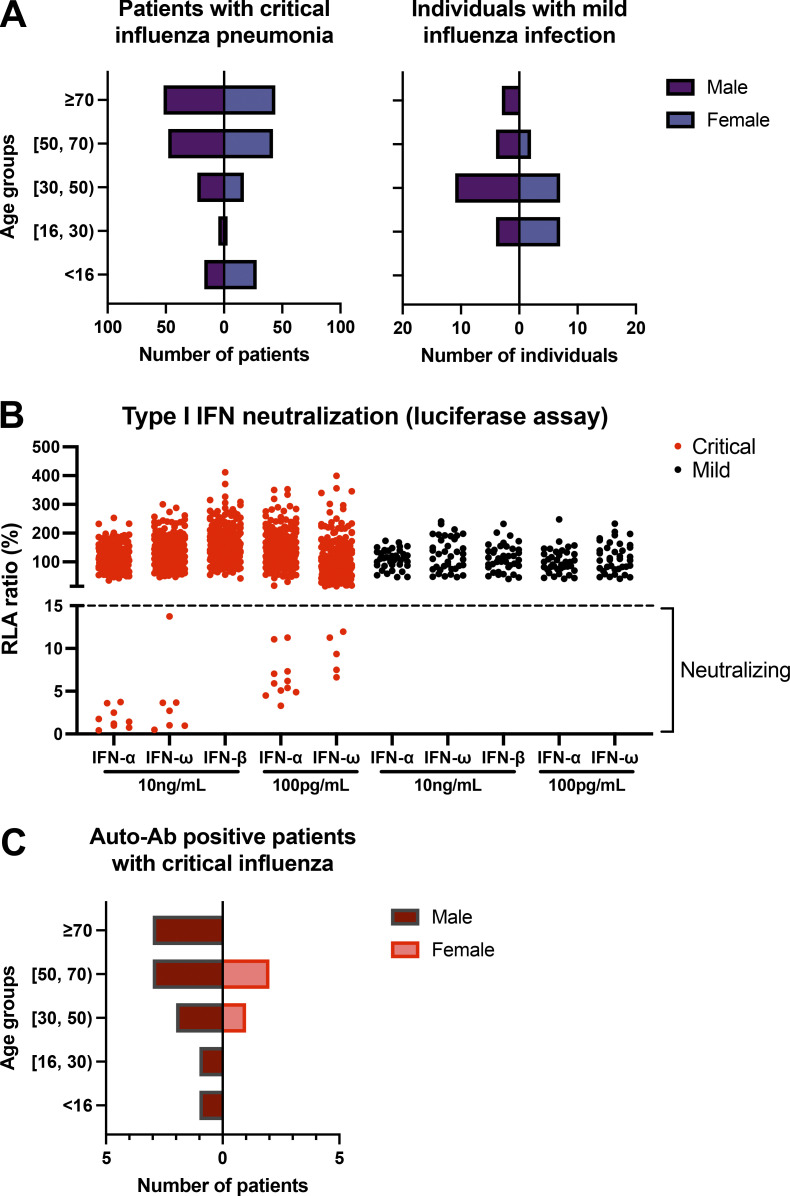
**Auto-Abs neutralizing IFN-α2 and/or IFN-ω in patients with critical influenza pneumonia. (A)** Age and sex distribution of the patients with critical influenza pneumonia or mild influenza infection. **(B)** Luciferase-based neutralization assay to detect auto-Abs neutralizing 10 ng/ml or 100 pg/ml IFN-α2, IFN-ω, or IFN-β. Plasma samples from patients with critical (red) or mild (black) influenza were diluted 1:10 in all tests. HEK293T cells were transfected with the dual luciferase system with IFN-sensitive response elements (ISRE) before treatment with type I IFNs with or without patient plasma, and relative luciferase activity (RLA) was calculated by normalizing firefly luciferase activity against *Renilla* luciferase activity. An RLA <15% of the value for the mock treatment was considered to correspond to neutralizing activity (dashed line; Bastard et al., 2021a). Experiments were repeated at least twice, and the average was plotted in the figure. **(C)** Age and sex distribution of patients with auto-Ab neutralizing IFN-α2 and/or IFN-ω (*n* = 13).

We recruited 279 patients from Belgium (31), Greece (5), Spain (40, including some cases described previously; Lopez-Rodriguez et al., 2016; Herrera-Ramos et al., 2014), Israel (1), and France (202) who had been hospitalized for critical influenza pneumonia, as defined by admission to an intensive care unit (ICU) for acute respiratory distress syndrome (ARDS) following a diagnosis of influenza and treatment with invasive or noninvasive mechanical ventilation or extracorporeal membrane oxygenation (ECMO), between 2012 and 2021. 32 of the 279 patients died, and 247 survived. The patients were between 7 d and 94 yr old; 52% were male and 48% were female (Fig. 1 A). We searched for circulating auto-Abs neutralizing type I IFNs in luciferase-based neutralization assays, as previously performed in patients with COVID-19 pneumonia and healthy donors (Bastard et al., 2021a). We identified 13 patients with neutralizing auto-Abs (P1–13), including 6 patients with auto-Abs neutralizing high concentrations (10 ng/ml) of both IFN-α2 and IFN-ω, 2 patients with auto-Abs neutralizing high concentrations of IFN-α2 and low concentrations (100 pg/ml) of IFN-ω, 1 patient with auto-Abs neutralizing high concentrations of IFN-α2 only, 1 patient with auto-Abs neutralizing low concentrations of both IFN-α2 and IFN-ω, and 3 patients with auto-Abs neutralizing low concentrations of IFN-α2 only (Table 1 and Fig. 1 B). None of the patients had auto-Abs neutralizing 10 ng/ml IFN-β. We previously showed that auto-Abs against IFN-α2 neutralized the other 12 forms of IFN-α (Bastard et al., 2020; Bastard et al., 2021a). Finally, we searched for auto-Abs against type III IFNs in 5 of the 13 patients with auto-Abs against type I IFNs. One of them (P3) had auto-Abs neutralizing IFN-λ1/IL-29 (half-maximal inhibitory concentration is 1:960 dilution for 12.5 pg/ml IL-29), but neither IFN-λ2 (IL-28A) nor IFN-λ3 (IL-28B; not depicted).

**Table 1.** Patients with auto-Abs neutralizing type I IFNs and influenza pneumonia

Patient	Auto-Abs					Gender	Age (yr)	Residence	Influenza pneumonia severity	Viral strain	Vaccinated	Clinical history	Outcome
	IFN-α (10 ng/ml)	IFN-α (100 pg/ml)	IFN-ω (10 ng/ml)	IFN-ω (100 pg/ml)	IFN-β (10 ng/ml)								
P1	+	+	+	+	−	M	73	Greece	Bilateral lung infiltrates, noninvasive mechanical ventilation	IAV (H3)	NA	COPD, sleep breathing disorder, cardiovascular disease, heart failure, hypertension, dyslipidemia	Survived
P2	+	NA	+	NA	−	F	67	Belgium	Admitted to ICU, noninvasive ventilation for 5 d	IAV	NA	Rheumatoid arthritis under methotrexate, local nasal corticosteroids	Survived
P3	+	+	+	+	−	M	28	Belgium	Admitted to ICU, invasive ventilation for 10 d	IAV	NA	Hypothyroidism, GI reflux, urolithiasis, limited metabolic syndrome (new-observation HbA1c 6.2%)	Survived
P4	+	+	+	+	−	F	56	Spain	ARDS	IAV (H1N1)	No		NA
P5	+	+	+	+	−	M	62	Spain	ARDS	NA	NA		NA
P6	+	+	+	+	−	M	62	France	Admitted to ICU, intubated	IAV	NA		Survived
P7	+	+	−	+	−	M	55	France	Admitted to ICU, intubated	IAV	NA		Survived
P8	+	+	−	−	−	M	70	France	Admitted to ICU, intubated	IAV	NA		Survived
P9	+	+	−	−	−	M	40	France	Admitted to ICU, ARDS, intubated, ECMO	IAV (H1N1)	NA	Meningitis at 3 mo of age	Survived
P10	−	+	−	+	−	M	6	Israel	Admitted to ICU, ARDS	IAV (H1N1)	No	Diagnosed with mitochondrial complex I deficiency (family history showed brother died of RSV infection at 3 mo of age)	Survived
P11	−	+	−	−	−	F	48	France	Admitted to ICU, intubated	IAV	NA		Survived
P12	−	+	−	−	−	M	39	France	Admitted to ICU, intubated, ECMO	IAV	NA		NA
P13	−	+	−	−	−	M	70	France	Admitted to ICU	IAV	NA		Survived
P14	+	+	+	+	+	M	55	Chile	Hospitalized with oxygen therapy	IAV	No	Dyslipidemia	Survived
P15	+	+	+	+	+	M	6	France	Intubated for hypoxemia influenza pneumonia, with secondary bacterial infection, intubated	IAV (H1N1)	NA	Failure to thrive, ulcerative digestive lesions due to disseminated CMV infection	Deceased
P16	+	+	+	+	+	F	1.3	Belgium	ARDS, intubated	IAV (H1N1)	No	Diarrhea after oral rotavirus vaccine and skin eruption after MMRV vaccine, autoimmune hemolytic anemia, autoimmune pancreatitis	Deceased
P17	+	+	+	+	−	M	43	Chile	Admitted to ICU, intubated	IAV (H1)	No	Chikungunya (2015) in Colombia	Survived
P18	+	+	+	+	−	M	64	Chile	Hospitalized with oxygen therapy	IAV (H3)	No	Dyslipidemia, infrarenal abdominal aortic aneurysm	Survived
P19	+	+	+	+	−	M	81	Chile	Hospitalized	IAV (H3)	Yes		Survived
P20	+	+	+	+	−	M	83	Spain	Hospitalized	IAV (H3)	Yes	Diabetes	Survived
P21	+	+	−	+	−	F	85	Chile	Admitted to ICU, intubated	IAV (H1)	No	Obesity, arterial hypertension, asthma	Survived
P22	+	+	−	−	−	F	91	Spain	Hospitalized with oxygen therapy	IAV (H1N1)	No	Asthma, heart disease, diabetes	Survived
P23	−	−	+	+	−	M	8	Taiwan	Admitted to ICU with oxygen therapy	IAV	NA	Sister died of encephalitis due to IAV (H1N1)	Survived

+, positive; −, negative; COPD, Chronic obstructive pulmonary disease; F, female; GI, gastrointestinal; M, male; NA, data not available; RSV, respiratory syncytial virus.

### Most patients with auto-Abs are male and <70 yr of age

The 13 auto-Ab–positive patients comprised 10 (77%) male patients and 3 female patients (23%); 1 of these patients was a child (<16 yr, 7.7%), 9 were adults aged 16–69 yr (69%), and 3 were elderly (≥70 yr old, 23%; Fig. 1 C). None of the auto-Ab–positive patients had been vaccinated against influenza in the year preceding disease onset. As in patients with critical COVID-19 and auto-Abs against type I IFNs (Bastard et al., 2021a; Bastard et al., 2020), the population of auto-Ab–positive patients with life-threatening influenza was mostly male, although this was not statistically significant. Indeed, 6.9% of male patients with critical influenza were auto-Ab positive, whereas only 2.2% of female patients with critical influenza were auto-Ab positive. The auto-Abs detected were of a similar nature to those observed in patients with critical COVID-19 pneumonia, with most patients having auto-Abs neutralizing high concentrations of IFN-α2 (∼70% in the influenza cohort and ∼60% in the COVID-19 cohort), a minority of patients having auto-Abs against IFN-ω only, and even fewer auto-Abs against IFN-β only (Table 1). We also recruited 38 patients with clinically diagnosed mild influenza infection who did not require hospitalization during the same period, including 14 from Spain and 24 from Greece. These patients were 18–80 yr old, and 57.9% were men. None of these 38 patients had auto-Abs neutralizing either high or low concentrations of IFN-α2, IFN-ω, or IFN-β (Fig. 1, A and B). Overall, auto-Abs neutralizing IFN-α2 alone or with IFN-ω were found in 4.7% of patients with life-threatening influenza pneumonia, 5.5% of patients <70 yr old, 6.9% of men with life-threatening influenza, and 7.5% of men <70 yr old.

### Individuals <70 yr old with auto-Abs against type I IFNs are at risk of critical influenza

**Figure 2.**
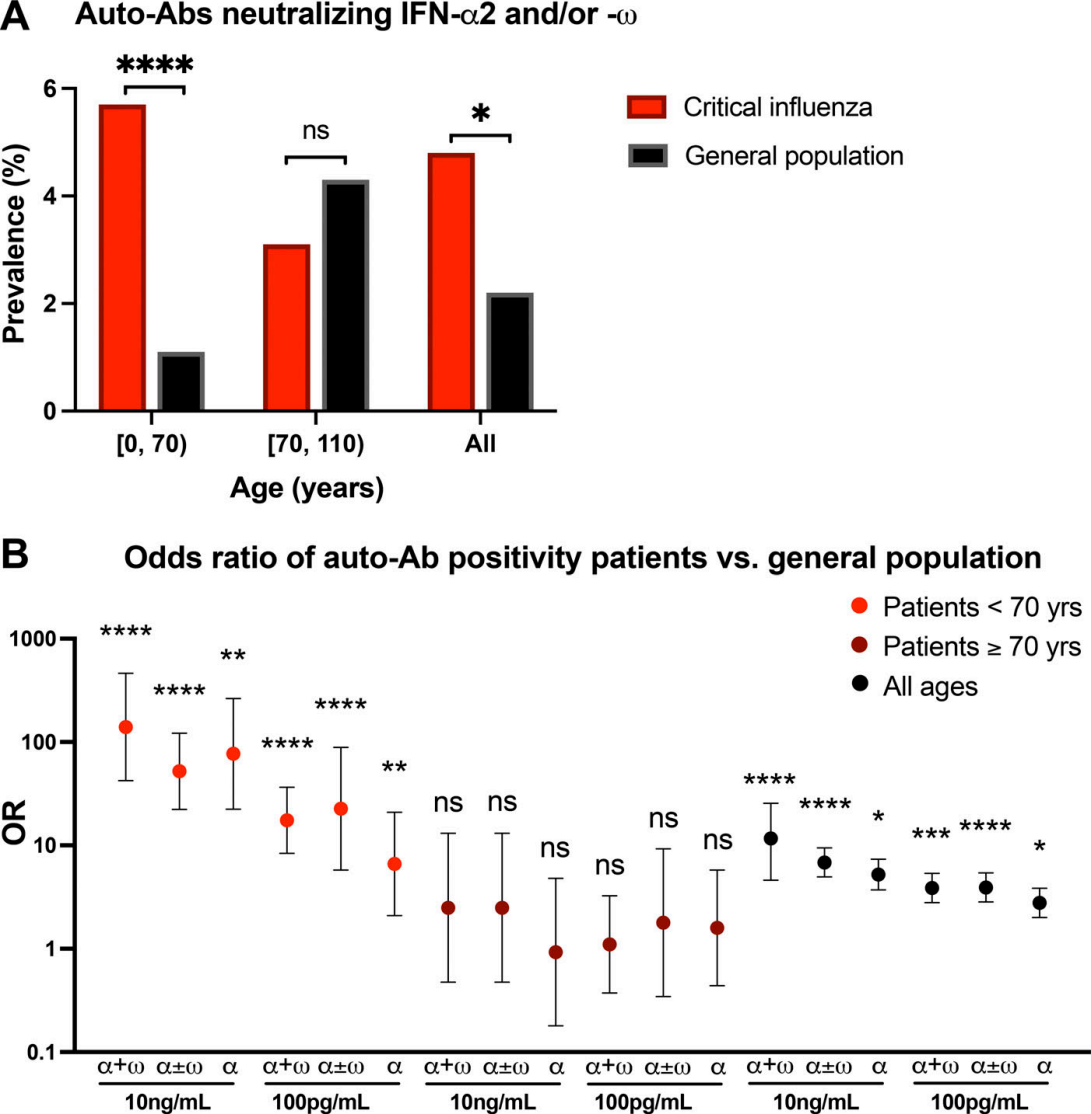
**Enrichment in auto-Ab–positive cases among patients with critical influenza pneumonia. (A)** Prevalence of auto-Ab–positive cases among patients with critical influenza (*n* = 279, red bars) and in the general population (*n* = 34,159, black bars). *, P < 0.05; ****, P < 10^−5^. **(B)** OR for the presence of auto-Abs, by sex and age, relative to the general population, with adjustment of the comparison by means of Firth’s bias-corrected logistic regression. The horizontal bars indicate the upper and lower limits of the 95% CIs. α + ω, auto-Abs neutralizing both IFN-α2 and IFN-ω; α ± ω, auto-Abs neutralizing IFN-α2 with or without IFN-ω; α, auto-Abs neutralizing IFN-α2 only; *, P < 0.05; **, P < 10^−2^; ***, P < 10^−3^; ****, P < 10^−4^.

We previously tested 34,159 healthy men and women aged 20–100 yr to estimate the prevalence of auto-Abs neutralizing type I IFNs in the uninfected general population (Bastard et al., 2021a). We further tested 1,065 healthy children, 12 (1.1%) of whom were found to be auto-Ab positive (Bastard et al., 2022b). We then compared the prevalence of auto-Abs against type I IFN between patients with life-threatening influenza and the general population. We first compared the prevalence of auto-Abs neutralizing at least low concentrations of IFN-α2 and/or IFN-ω, which were present in the largest number of patients (13 carriers among the 272 individuals tested, 4.8%) and members of the general population, in a sex- and age-adjusted Firth’s bias-corrected logistic regression analysis. We found a general enrichment in these auto-Abs in patients with critical influenza relative to the general population (2.2%; odds ratio [OR] = 2.3, 95% confidence interval [CI] 1.2–3.9, P = 0.01; Fig. 2 A). We then investigated the age effect in greater detail. We found a significant interaction between age, classified into two groups (younger individuals <70 yr old, and older individuals ≥70 yr old), and the presence of these auto-Abs. Indeed, the prevalence of auto-Abs was significantly higher in younger patients (5.7 vs. 1.1%, OR = 5.7, 95% CI 3.0–11.1, P = 2.2 × 10^−5^) with critical influenza than in the general population, whereas no significant enrichment was observed in older patients (3.1 vs. 4.4%, OR = 0.80, 95% CI 0.27–2.4, P = 0.68), consistent with the distribution of auto-Ab prevalence across age groups (Fig. 2 A). In summary, these results suggest that auto-Ab–positive individuals <70 yr of age have a higher risk of developing critical influenza pneumonia than auto-Ab–negative individuals.

### Risk of critical influenza according to the nature of the auto-Abs against type I IFNs

We then performed the same logistic regression analyses taking into account all combinations of auto-Abs based on the nature and concentration of type I IFNs neutralized. All combinations of auto-Abs neutralizing different concentrations of IFN-α2, with or without IFN-ω, were significantly associated with critical influenza, albeit to different extents (Fig. 2 B). The presence of auto-Abs neutralizing high concentrations of both IFN-α2 and IFN-ω was associated with the highest risk of developing critical influenza in the overall sample (OR = 11.7, 95% CI 4.6–25.5, P = 1.3 × 10^−5^). The presence of auto-Abs neutralizing high concentrations of IFN-α2 only, low concentrations of both IFN-α2 and IFN-ω, or IFN-α2 only was associated with a three to five times higher risk of developing critical influenza (Fig. 2 B). Furthermore, the presence of auto-Abs neutralizing high concentrations of both IFN-α2 and IFN-ω had an even stronger impact in the subsample of subjects <70 yr old (OR = 139.9, 95% CI 42.3–462.5, P = 3.1 × 10^−10^), and this effect was even more marked in men <70 yr old (OR = 167.3, 95% CI 33.3–840.2, P = 3.2 × 10^−7^). The presence of auto-Abs neutralizing high concentrations of IFN-α2 only or low concentrations of both IFN-α2 and IFN-ω resulted in a 20–80 times higher risk of developing critical influenza in patients <70 yr, whereas the presence of auto-Abs neutralizing low concentrations of IFN-α2 only resulted in an almost seven times higher risk of developing critical influenza in patients <70 yr (Fig. 2 B). We identified no patients with auto-Abs neutralizing IFN-ω only or IFN-β, whereas these antibodies were found in 1.4 and 0.2% of the general population, respectively (Bastard et al., 2021a). The absence of such antibodies in the patients in our sample was probably due to the small size of the sample tested, but this finding nevertheless suggests that the presence of such antibodies in the general population does not confer a strong predisposition to critical influenza, if, indeed, it increases susceptibility at all. In summary, the risk of critical influenza increased with both the concentration and number of type I IFNs neutralized by the auto-Abs. These findings are consistent with those previously reported for patients with critical COVID-19 pneumonia (Bastard et al., 2021a).

### The auto-Abs neutralized the antiviral function of type I IFNs in respiratory epithelial-like A549 cells infected with IAV

**Figure 3.**
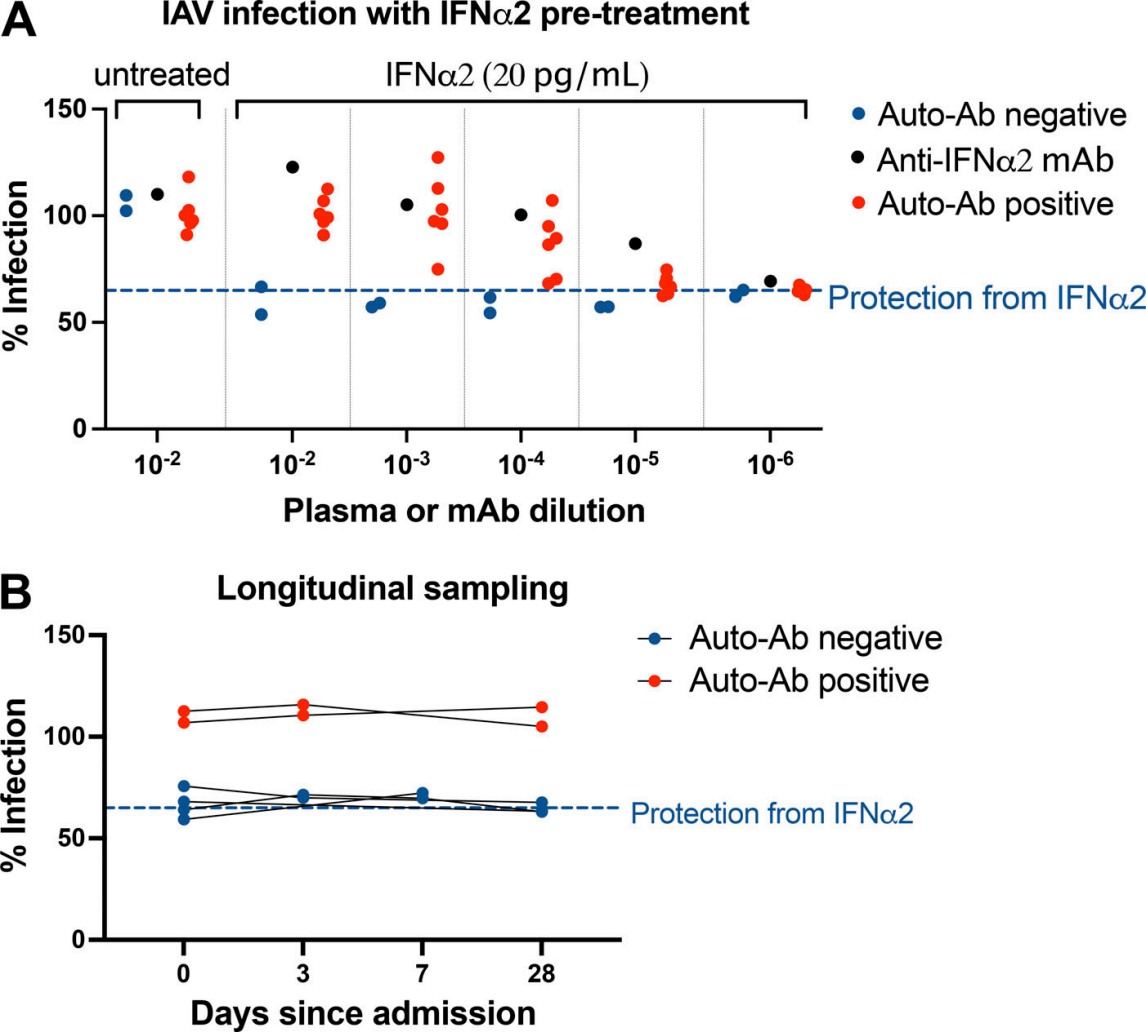
**Neutralizing auto-Abs block the antiviral function of IFN-α2 in IAV-infected A549 epithelial cells. (A)** A549 cells were treated with 20 pg/ml exogenous IFN-α2 with or without patient plasma (titrated to the dilutions indicated on the x axis), anti–IFN-α2 monoclonal antibody, and healthy donor plasma overnight before infection with IAV Cal/09 virus expressing NS1-mCherry (CalNSmCherry) at an MOI of 0.5. The day after infection, the percentage of the cells infected was determined with a Celigo (Nexcelcom) imaging cytometer. The dotted line at 64.98% represents the mean percentage infection in cells treated with 20 pg/ml IFN-α2 in the absence of plasma or anti–IFN-α2 antibody. Experiments were repeated four times. **(B)** Longitudinal testing of six patients with life-threatening influenza pneumonia (two positive and four negative for auto-Abs), with the assay as described in A.

These findings suggested that auto-Abs might block the antiviral activity of type I IFNs against IAV in vivo. We tested this hypothesis by subjecting an REC line, A549, to pretreatment with IFN-α2, with or without patient plasma, before infecting the cells with IAV (Cal/09 virus expressing mCherry). We then determined the percentage of mCherry-positive cells with an imaging cytometer, as an indicator of IAV replication (shown as percentage infection). Serial titration showed that the minimum concentration of IFN-α2 required for robust antiviral activity was 20 pg/ml, which blocked ∼40% of IAV infection. We found that plasma from auto-Ab–positive patients completely neutralized 20 pg/ml IFN-α2 at a dilution of 1:100, as shown by the IAV infection rate of 100% (Fig. 3 A). We then further diluted the patients’ plasma to titrate neutralization capacity. We found that, when diluted 1:10,000, plasma from four of the six patients tested still effectively blocked the antiviral activity of 20 pg/ml IFN-α2 (Fig. 3 A). Thus, the auto-Abs from the patients tested blocked the anti-IAV activity of type I IFNs in vitro, thereby facilitating viral replication. These findings also indicate that some patients have auto-Abs with such high titers and/or affinity that they can block the antiviral activity of type I IFNs at concentrations beyond the physiological range (>20 pg/ml). Overall, we found that auto-Abs against type I IFNs from patients with critical influenza pneumonia neutralized the protective function of type I IFNs against IAV in vitro. For six patients (two auto-Ab–positive and four auto-Ab–negative) from whom plasma samples were collected at multiple time points, we found that neutralization capacity remained stable for ≥4 wk after admission (Fig. 3 B), consistent with previous observations in patients with life-threatening COVID-19 and auto-Abs neutralizing type I IFNs (van der Wijst et al., 2021; Shaw et al., 2021).

### Neutralizing auto-Abs block the antiviral function of type I IFNs in reconstituted human airway epithelia (HAE) infected with IAV

**Figure 4.**
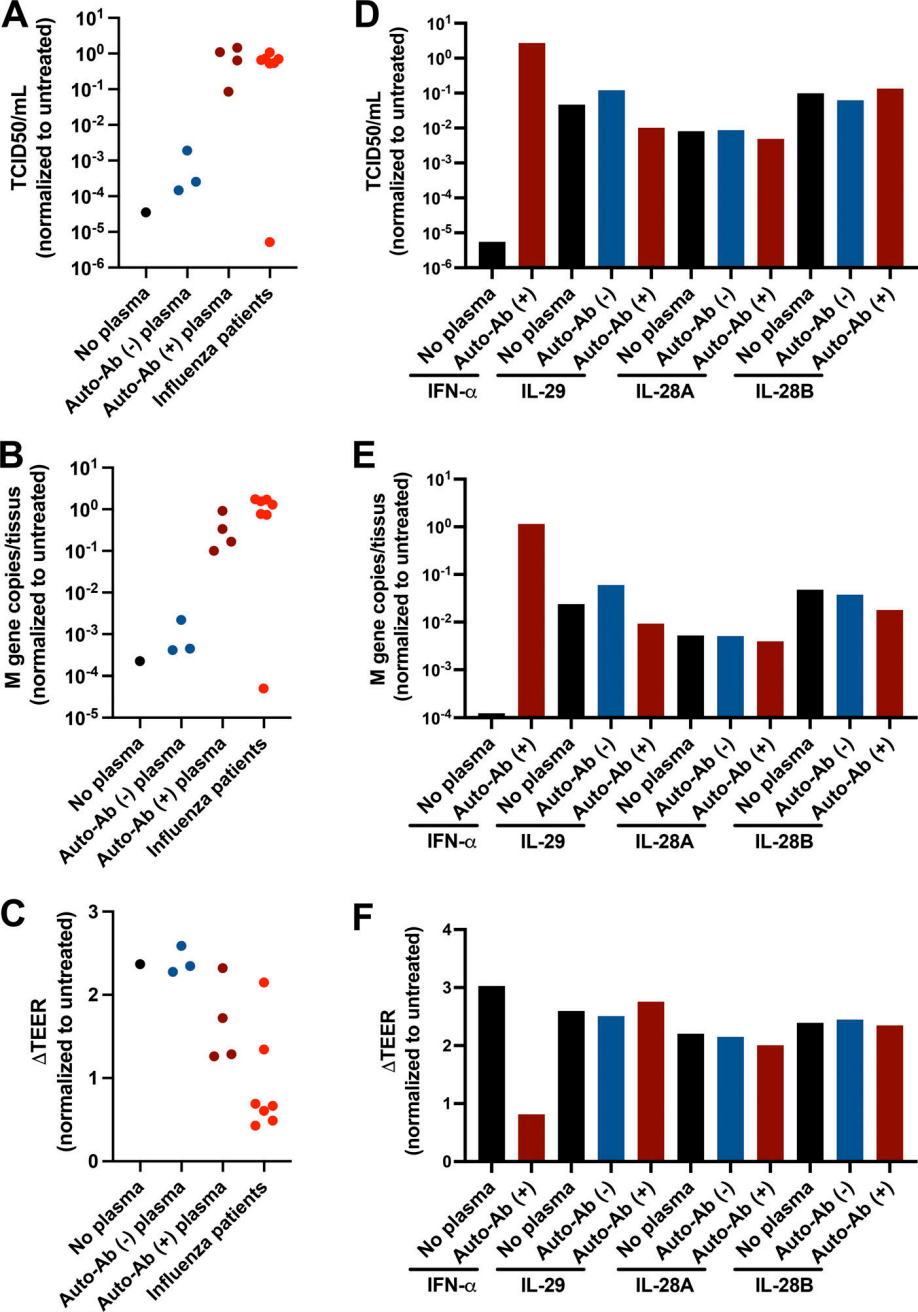
**Neutralizing auto-Abs block the antiviral function of IFN-α2 in IAV-infected HAE cultures. (A–F)** HAE reconstituted from human nasal primary cells and maintained in an air–liquid interface were either left untreated or treated with 2 ng/ml exogenous IFN-α2a (A–C) or 20 ng/ml exogenous IL-29, IL-28A, or IL-28B (D–F), in the presence of inactivated patient plasma (1:100 diluted) for 24 h before IAV infection. Cells were treated again on the basolateral side with same concentration of IFN-α2a or IFN-λ1/IL-29, IFN-λ2/IL-28A, or IFN-λ3/IL-28B in the presence of patient plasma (*n* = 7) 1 h after IAV infection. These seven patients had auto-Abs neutralizing IFN-α at 10 ng/ml, but not IFN-β or -λ. HAE apical poles were washed, 54 h after infection, and titrated by TCID_50_ determination (A and D) and quantitative RT-PCR (B and E). Changes in TEER (ΔTEER) were measured as a surrogate for the integrity of HAE (C and F). Previously identified auto-Ab–positive (auto-Ab [+]) or auto-Ab [−] plasma samples were used as controls. Experiments were repeated three times.

**Figure 5.**
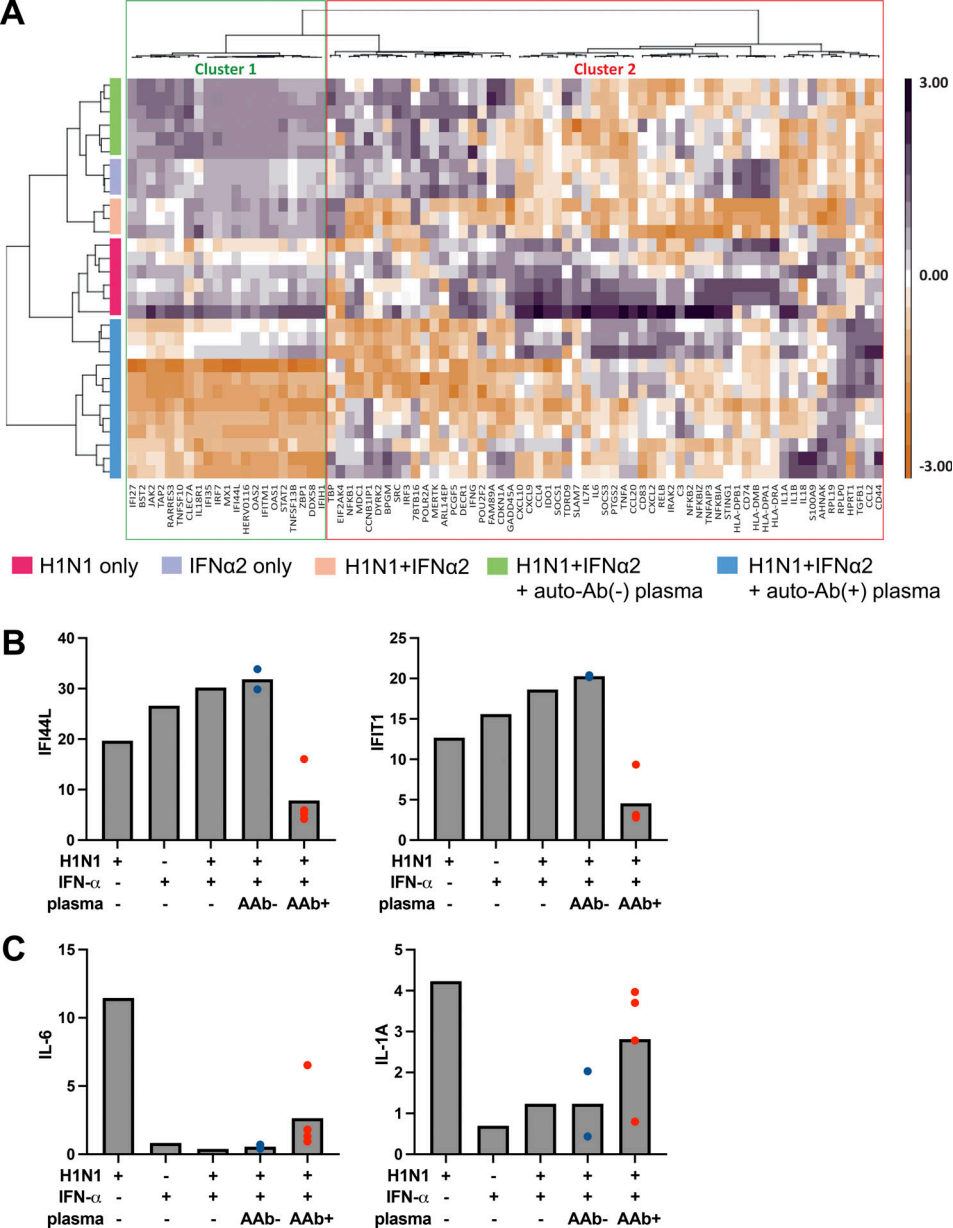
**ISG and proinflammatory responses in IAV-infected HAE cultures.** HAE reconstituted from human nasal primary cells and maintained in an air–liquid interface were either left untreated or treated with 2 ng/ml exogenous IFN-α2a in the presence of inactivated patient plasma (1:100 dilution) for 24 h before IAV infection. Cells were treated again on the basolateral side with 2 ng/ml IFN-α2a in the presence of patient plasma 1 h after IAV infection. RNA was isolated 54 h after infection, and NanoString analysis was performed with a panel of immune response genes. **(A)** Heatmap of gene expression profiles from unsupervised analysis (Euclidean distance matrix, Ward’s method) generated by scaling and centering log_10_-transformed normalized gene expression (expressed as fold-change induction relative to mock conditions) and based on the full 96-gene panel. Gene and sample clustering is indicated by dendrogram trees above and to the left, respectively, of the heatmap. Gene clustering distinguished ISGs (cluster 1) from proinflammatory genes (cluster 2; Table S1). **(B and C)** Relative expression (mean) levels of two ISGs, IFI44L and IFIT1 (B), and two proinflammatory cytokines, IL-6 and IL1A (C), based on NanoString analysis on total cellular RNA extracted after infection. Gene expression is expressed as a fold-change induction relative to mock conditions (untreated/uninfected). AAb−, auto-Ab–negative plasma; AAb+, auto-Ab–positive plasma.

We tested the hypothesis that auto-Abs block the antiviral activity of type I IFNs against IAV in HAE grown in an air–liquid interface, which mimics the physiological environment for IAV and SARS-CoV-2 infections in primary human cells (Pizzorno et al., 2019; Pizzorno et al., 2020). We treated HAE cells with 2 ng/ml IFN-α2 (24 h before and 1 h after IAV infection) in the presence or absence of patient plasma (1:100 dilution) and infected the cells with IAV (H1N1 pdm09). IFN-α2 strongly inhibited viral replication, as indicated by the 50% tissue culture infectious dose (TCID_50_) and M gene copy numbers (Fig. 4, A and B). We tested plasma from seven patients with critical influenza and auto-Abs neutralizing 10 ng/ml IFN-α in luciferase assays at a 1:10 dilution. Plasma from six of the seven patients blocked the antiviral activity of 2 ng/ml IFN-α2 at a 1:100 dilution. More importantly, IAV infection led to a decrease in transepithelial electrical resistance (TEER), a measurement of the integrity of the epithelial barrier. IFN-α2 treatment can protect the epithelial barrier from IAV, thereby maintaining TEER. Plasma from five of the seven patients with severe influenza tested blocked the protective function of IFN-α2 (Fig. 4 C). Thus, IAV replication is associated with a loss of epithelial integrity, whereas type I IFN treatment is not. We also tested type III IFNs, including IFN-λ1 (IL-29), -λ2 (IL-28A), and -λ3 (IL-28B), in the same HAE system. We showed that 20 ng/ml type III IFNs also inhibited IAV replication and maintained normal TEER, although less effectively than IFN-α2 (Fig. 4, D and E). Interestingly, plasma from two patients did not block the protective function of IFN-α2, and one of these plasma samples did not block the antiviral activity of 2 ng/ml IFN-α2 completely when diluted 1:100 (Fig. 4, A–C). This was probably due to the high dilution of plasma in the HAE culture, to minimize nonspecific inhibition and toxicity due to the presence of human plasma. This observation may also suggest that differentiated RECs in the air–liquid interface and HEK293T cells respond differently in neutralization assays. We thus studied the biological consequences of auto-Abs in the HAE system with a Nanostring hybridization-based assay for multiplex mRNA detection and relative quantification for a panel of immune response genes. We found that IFN-α2 treatment or IAV infection induced the expression of IFN-stimulated genes (ISGs) in HAEs, and that this expression was blocked by auto-Ab–positive plasma from patients (Fig. 5, A and B). Consistent with TEER measurements, the levels of proinflammatory cytokines, including IL-6 and IL-1A, were higher in the presence of auto-Abs and high viral titers, further suggesting that viral replication led to epithelial damage and inflammation (Fig. 5 C). The levels of auto-Abs in the blood are correlated with, but not identical to, those in the respiratory tract (Lopez et al., 2021; Ziegler et al., 2021; Zhang et al., 2022), and the patient plasma used in the HAE culture was diluted 1:100, whereas a dilution of 1:10 was used for neutralization assays with HEK cells. In summary, the auto-Abs found in the patients with critical influenza blocked the antiviral function of IFN-α2 in respiratory models in vitro, increasing viral replication and tissue damage, together with the production of proinflammatory cytokines by damaged cells.

### Auto-Abs neutralizing IFN-α2 and/or IFN-ω in five additional cohorts of patients hospitalized with influenza pneumonia

**Figure 6.**
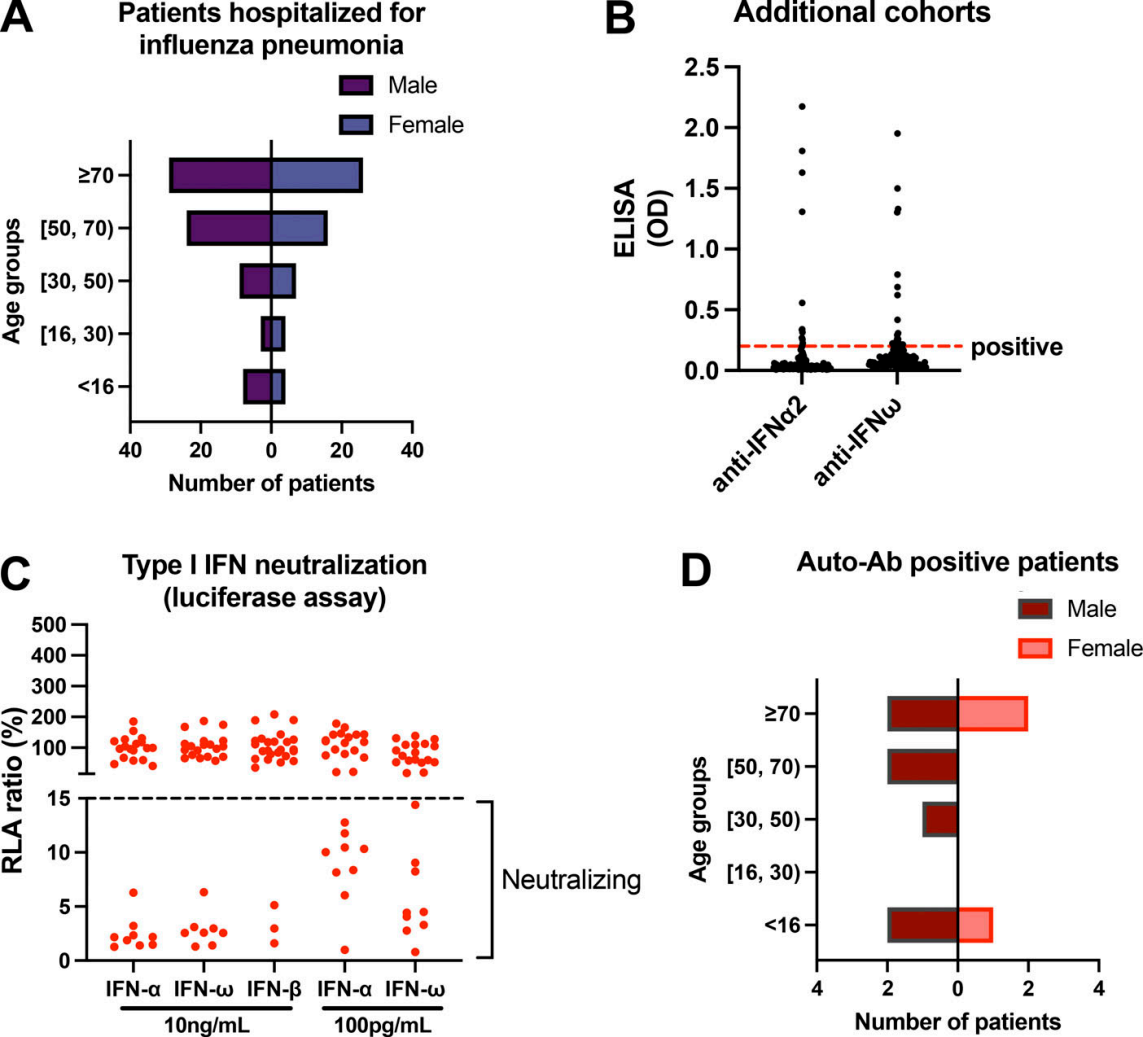
**Auto-Abs neutralizing IFN-α2 and/or IFN-ω in ELISA-positive patients hospitalized with influenza pneumonia in additional cohorts. (A)** Age and sex distribution of patients from Chile, Spain, France, Belgium, and Taiwan hospitalized for influenza pneumonia (*n* = 130). **(B)** Patient plasma samples were tested by ELISA for auto-Abs against IFN-α2 and -ω. Patient plasma samples were diluted 1:50 before being added to plates coated with 2 μg/ml rhIFN-α or rhIFN-ω. HRP-conjugated goat antiserum against human IgG or IgA was added to final concentration of 2 μg/ml. OD was measured. Each plasma sample was tested once. **(C)** Luciferase-based neutralization assay to detect auto-Abs neutralizing 10 ng/ml or 100 pg/ml IFN-α2, IFN-ω, or IFN-β. Plasma samples from ELISA-positive patients were diluted 1:10 in all tests. HEK293T cells were transfected with the dual luciferase system with IFN-sensitive response elements (ISRE) before treatment with type I IFNs with or without plasma from patients, and relative luciferase activity (RLA) was calculated by normalizing firefly luciferase activity against *Renilla* luciferase activity. An RLA <15% the value for the mock treatment was considered to indicate that the antibodies were neutralizing (dashed line). **(D)** Age and sex distribution of patients with auto-Ab neutralizing IFN-α2 and/or IFN-ω (*n* = 10).

In five other independent cohorts of 130 patients hospitalized for influenza pneumonia from Chile (82), Spain (45), France (1), Belgium (1), and Taiwan (1), including 84 patients requiring oxygen therapy (65%), screening for auto-Abs was performed by ELISA rather than neutralization assays (Fig. 6, A and B). Auto-Abs against IFN-α2 and/or IFN-ω were detected by ELISA in 27 patients (20%) aged 1–97 yr (65% of whom were male), including 15 patients requiring oxygen therapy. Because of the limited volumes of sample available, we tested only plasma samples from these ELISA-positive patients in our luciferase-based neutralization assays (Fig. 6 B). Only 10 of the 27 patients had neutralizing auto-Abs (P14–23): 3 with auto-Abs neutralizing high concentrations of IFN-α2, IFN-ω, and IFN-β (P14–16), 4 with auto-Abs neutralizing high concentrations of IFN-α2 and IFN-ω (P17–20), 1 with auto-Abs neutralizing high concentrations of IFN-α2 and low concentrations of IFN-ω (P21), 1 with auto-Abs neutralizing high concentrations of IFN-α2 only (P22), and 1 with auto-Abs neutralizing high concentrations of IFN-ω only (P23; Fig. 6 C and Table 1). 8 of the 10 patients required oxygen, including 4 intubated and ventilated and 1 who died from critical pneumonia (P15). Overall, 7.7% of the patients in these 5 cohorts were found to have auto-Abs neutralizing IFN-α2 and/or IFN-ω. The 10 patients included 3 children (30%), 3 adults under <70 yr old (30%), and 4 elderly patients (40%); 7 of the patients were male (70%; Fig. 6 D). Like the 13 patients identified in the previous cohort, an enrichment in male patients was observed among patients with neutralizing auto-Abs. However, there were more elderly patients in these additional cohorts, probably owing to a recruitment bias (Fig. 6, A and D; and Table 1). These data further suggest that auto-Abs against type I IFN are associated with influenza pneumonia. They also suggest that ELISA-based assays can be used as a screening method, albeit of limited diagnostic value and with many more false positives than neutralization. Furthermore, some ELISA-negative cases may actually have neutralizing auto-Abs (not tested here), thereby constituting false negatives, as shown in our previous study of COVID-19 (Bastard et al., 2021a). The results from these five additional cohorts cannot be used for the calculation of prevalence or ORs due to the lack of screening by neutralization, but they add weight to the notion that auto-Abs against type I IFNs increase susceptibility to hypoxemic influenza pneumonia.

## Discussion

We found that almost 5% of patients with critical influenza pneumonia studied internationally had auto-Abs neutralizing IFN-α2 alone or with IFN-ω. We showed that these auto-Abs neutralized 10 ng/ml or at least 100 pg/ml type I IFNs in plasma diluted 1:10. The population of patients with critical influenza pneumonia was significantly enriched in auto-Ab–positive cases relative to a small sample of individuals with mild influenza infection or a much larger sample of individuals from the general population. The neutralizing auto-Abs blocked the antiviral activity of 20 pg/ml IFN-α2 in A549 cells infected with IAV, even when diluted 1:1,000. They also blocked the antiviral activity of IFN-α2 in HAEs infected with IAV in vitro, further suggesting that the auto-Abs were detrimental in IAV-infected human RECs in vivo. We showed that auto-Abs neutralizing IFN-α2 alone or with IFN-ω were present in almost 5% of patients with life-threatening influenza pneumonia, including ∼6% of patients <70 yr old, ∼7% of men, and ∼8% of men <70 yr old. Auto-Abs neutralizing type I IFNs can also underlie life-threatening COVID-19 pneumonia and severe adverse reactions to the live attenuated yellow fever virus vaccine (Pozzetto et al., 1984; Bastard et al., 2020, 2021a, 2021c, Casanova and Abel, 2021b, 2022; Goncalves et al., 2021; Lopez et al., 2021; Zhang et al., 2022). Notably, the discovery of AR inborn errors of type I and III IFN immunity in patients with life-threatening influenza pneumonia (mutations of *IRF7*, *STAT2*, and *IRF9*; Ciancanelli et al., 2015; Hernandez et al., 2018; Lim et al., 2019; Freij et al., 2020) led to that of overlapping (IRF7) and other (IFNAR1) AR etiologies of critical COVID-19 pneumonia (Asano et al., 2021; Zhang et al., 2020b), and conversely, the discovery of AR IFNAR1 deficiency and auto-Abs against type I IFNs in patients with critical COVID-19 pneumonia (Bastard et al., 2020; Bastard et al., 2021a) led to that of auto-Abs against type I IFNs in patients with critical influenza. It is intriguing that the known patients with AR IFNAR1 or IFNAR2 deficiency did not suffer from critical influenza (Bastard et al., 2022a; Hernandez et al., 2019; Bastard et al., 2021b; Duncan et al., 2022; Duncan et al., 2015). This may reflect the small number of patients diagnosed, their previous viral illnesses (e.g., MMR disease), prompting influenza vaccination, and an ascertainment bias. Our findings suggest that IFNAR1- or IFNAR2-deficient patients may be prone to critical influenza.

The greater enrichment in patients with auto-Abs against type I IFNs among patients with critical COVID-19 than among those with critical influenza is also intriguing. Indeed, although individuals with auto-Abs neutralizing these type I IFNs are 3–12 times more likely overall to develop critical influenza pneumonia than the general population, the overall prevalence of these auto-Abs in patients with critical influenza pneumonia is close to 5%, a figure significantly lower than the 15% of critical COVID-19 pneumonia patients with these antibodies (Bastard et al., 2021a). This observation may reflect the higher virulence of SARS-CoV-2 than of seasonal influenza viruses in unvaccinated individuals. We can speculate that a value of 15% would have been found among patients with critical influenza due to the 1918 H1N1 virus or in other, more recent influenza pandemics (Reichert et al., 2012; Krammer et al., 2018). We can also speculate that the lower the induction of type I IFNs by the virus, the higher the virulence, and the greater the vulnerability of individuals with auto-Abs against type I IFNs or with IFNAR1 or IFNAR2 deficiencies or other inborn errors of type I IFN immunity (Chen et al., 2021). Moreover, previous anti-influenza vaccination or infections with one or more related influenza viruses may mitigate the clinical impact of infections with new viral strains, including those in patients with auto-Abs against type I IFNs. The age-stratified analysis of our data supports this hypothesis. Indeed, we showed an enrichment in auto-Ab–positive cases among patients <70 yr of age, but not in older patients. Auto-Abs against type I IFNs were found in only three sick children, consistent with previous observations that pathogenic auto-Abs against type I IFNs are rare in children (Bastard et al., 2021a). Moreover, a strong enrichment in auto-Ab–positive cases was observed for younger patients (<70 yr old), with an OR of ∼7–140 depending on the nature of the auto-Abs, but not for older patients. The same trend was observed in patients with critical COVID-19 pneumonia (Manry et al., 2022), suggesting that risk factors other than auto-Abs contribute to critical influenza in the elderly.

Finally, our data highlight the major impact of the nature and concentration of type I IFNs neutralized by circulating auto-Abs on the risk of developing critical influenza pneumonia, as previously shown for COVID-19 pneumonia (Bastard et al., 2021a). After adjustment for age and sex, patients with auto-Abs neutralizing high concentrations of both IFN-α2 and IFN-ω were found to have the highest risk of critical influenza pneumonia (OR = 139.9 in patients <70 yr old, OR = 11.7 for all ages), whereas patients with auto-Abs neutralizing low concentrations IFN-α presented a smaller increase in the risk of critical pneumonia (OR = 6.6 in patients <70 yr old, OR = 2.8 for all ages). We identified only one patient with auto-Abs neutralizing high concentrations of IFN-ω only, and such antibodies were also rare in patients with critical COVID-19 pneumonia (0.8%; Bastard et al., 2021a). We found no patients with auto-Abs neutralizing IFN-β only, whereas such antibodies were found in almost 1% of patients with critical COVID-19 pneumonia (Bastard et al., 2021a). All auto-Abs neutralizing IFN-α2 also neutralize the other 12 subtypes of IFN-α, but auto-Abs neutralizing IFN-ω or IFN-β neutralize only a single subtype of IFN (Bastard et al., 2021a; Bastard et al., 2020), making it less likely that such antibodies underlie critical seasonal influenza. It would be interesting to screen patients with critical influenza for auto-Abs neutralizing type III IFNs. These auto-Abs might contribute to influenza and other severe viral infections, especially of the respiratory tract (Lim et al., 2019). Overall, auto-Abs neutralizing type I IFNs can underlie at least three severe viral diseases, with an apparently greater risk of critical COVID-19 pneumonia than of critical influenza, while the risk of yellow fever vaccine disease is more difficult to estimate, given the small number of patients tested. These auto-Abs may also underlie other viral diseases, including severe disease caused by the varicella zoster virus, as disseminated zoster was the clinical manifestation of the first patient with causal auto-Abs against type I IFN ever described, by Ion Gresser in 1984 (Pozzetto et al., 1984; Walter et al., 2015; Busnadiego et al., 2022; Mathian et al., 2022).

## Materials and methods

### Patients

We recruited 279 patients from Belgium (31), Greece (5), Spain (40, including some cases described previously; Lopez-Rodriguez et al., 2016; Herrera-Ramos et al., 2014), Israel (1), and France (202) who had been hospitalized for critical influenza pneumonia, as defined by admission to an ICU for ARDS following a diagnosis of influenza and treatment with invasive or noninvasive mechanical ventilation or ECMO between 2012 and 2021. From the same clinical centers in Greece (24) and Spain (14), we recruited patients with mild influenza infections not requiring hospitalization during the same period. In addition, we recruited five other independent cohorts of 130 patients hospitalized for influenza pneumonia from Chile (82), Spain (45), France (1), Belgium (1), and Taiwan (1), including 84 patients requiring oxygen therapy (65%). All the patients were diagnosed with influenza infection by PCR. We previously tested 34,159 healthy men and women aged 20–100 yr to estimate the prevalence of auto-Abs neutralizing type I IFNs in the uninfected general population (Bastard et al., 2021a). We further tested 1,065 healthy children, 12 of whom (1.1%) were found to be auto-Ab–positive (Bastard et al., 2022b). Written informed consent was obtained in the country of residence of the patients, in accordance with local regulations, and with institutional review board approval. Experiments were conducted in the United States, France, and Estonia in accordance with local regulations and with the approval of the institutional review board. Approval was obtained from the French Ethics Committee “Comité de Protection des Personnes,” the French National Agency for Medicine and Health Product Safety, the “Institut National de la Santé et de la Recherche Médicale,” in Paris, France (protocol no. C10-13), and the Rockefeller University Institutional Review Board in New York, NY (protocol no. JCA-0700). For the Chilean samples, clinical and epidemiological data and the corresponding clinical specimens were collected after informed written consent was obtained under protocol 16-066, which was reviewed and approved by the Scientific Ethics Committee for Health Sciences (CECSaludUC) at Pontificia Universidad Católica de Chile.

### Luciferase reporter assays

The blocking activity of anti–IFN-α2 and anti–IFN-ω auto-Abs was determined with a reporter luciferase assay. Briefly, HEK293T cells were transfected with a plasmid containing the firefly luciferase gene under the control of the human *ISRE* promoter in the pGL4.45 backbone and a plasmid constitutively expressing the *Renilla* luciferase for normalization (pRL-SV40). Cells were transfected by incubation for 24 h with the plasmids and X-tremeGene9 transfection reagent (ref. number 6365779001; Sigma-Aldrich). Cells in DMEM (Thermo Fisher Scientific) supplemented with 2% FCS and 10% healthy control or patient serum/plasma (after inactivation at 56°C, for 20 min) either were left unstimulated or were stimulated with IFN-α2 (ref. number 130-108-984; Miltenyi Biotec) or IFN-ω (ref. number SRP3061; Merck), at a concentration of 10 ng/ml or 100 pg/ml, or IFN-β (ref. number 130-107-888; Miltenyi Biotech) at 10 ng/ml, for 16 h at 37°C. Each sample was tested once for each cytokine and dose. Finally, cells were lysed for 20 min at room temperature, and luciferase levels were measured with the Dual-Luciferase Reporter 1000 assay system (ref. number E1980; Promega), according to the manufacturer’s protocol. Luminescence intensity was measured with a VICTOR-X Multilabel Plate Reader (PerkinElmer Life Sciences). Firefly luciferase activity values were normalized against *Renilla* luciferase activity values. These values were then normalized against the median induction level for nonneutralizing samples and expressed as a percentage. Samples were considered neutralizing if luciferase induction, normalized against *Renilla* luciferase activity, was <15% of the median value for controls tested the same day. For 35 patients whose plasma did not neutralize 100 pg/ml IFN-α2 or IFN-ω, we did not perform the neutralization assay with 10 ng/ml IFN-α2/-ω due to the limited volume of plasma available.

### Functional evaluation of IFN auto-Abs

A549 cells (CRM-CCL-185; ATTC) were cultured in DMEM (Gibco) supplemented with 10% FBS (PEAK) and penicillin-streptomycin (Gibco), at 37°C, under an atmosphere containing 5% CO_2_. Cells were tested periodically for mycoplasma contamination, with negative results in all cases.

A549 cells were used to seed 96-well plates at a density of 3 × 10^3^ cells/well. The next day, a commercial anti–IFN-α2 antibody (catalog number 21100-1; R&D Systems) of plasma samples were serially diluted (10-fold) and incubated with 20 pg/ml recombinant IFN-α2 (catalog number 11101-2; R&D Systems) for 1 h at 37°C (starting concentration: plasma samples = 1/100 and anti–IFN-α2 antibody = 1/100). The cell culture medium was then removed from the 96-well plates and replaced with the plasma/antibody–IFN-α2 mixture. Each sample was tested once, in triplicate. The plates were incubated overnight, and the plasma/antibody–IFN-α2 mixture was removed by aspiration. The cells were then washed three times with PBS to remove potential anti-influenza neutralizing antibodies and infected with a recombinant Cal/09 virus expressing NS1-mCherry (CalNSmCherry) at a multiplicity of infection (MOI) of 0.5. 16 h after infection, cells were fixed with 4% formaldehyde, washed twice with PBS, and stained with DAPI. The percentage of infected cells was quantified with a Celigo (Nexcelcom) imaging cytometer.

### HAE infection with IAV

#### Influenza seroneutralization assay in Madin-Darby canine kidney cells

Plasma samples were serially diluted in MEM (Lonza) supplemented with 2 mM L-glutamine (Gibco), 100 U/ml penicillin, 100 µg/ml streptomycin (Gibco), and 1 µg/ml acetylated trypsin from bovine pancreas (Sigma-Aldrich). Serial dilutions were mixed with 100 TCID_50_ of A/Lyon/969/2009 H1N1 virus and incubated at 37°C for 1 h. We then inoculated 96-well plates containing confluent Madin-Darby canine kidney cells in 150 μl of supplemented MEM in quadruplicate with 50 μl per well of the plasma-virus dilutions and incubated the plates at 37°C, under an atmosphere containing 5% CO_2_. After 96 h of incubation, we checked for cytopathic effects by microscopy. The anti-influenza seroneutralization titer for each plasma sample is expressed as the inverse of the highest dilution at which no cytopathic effects were observed in at least two of the four wells.

#### Viral infection and IFN treatment in reconstituted HAE

MucilAir HAE reconstituted from human nasal primary cells (pool of 14 donors with no identified diseases) were provided by Epithelix SARL and maintained in an air–liquid interface at 37°C, under an atmosphere containing 5% CO_2_, in specific culture medium in Costar Transwell inserts according to the manufacturer’s instructions. The day before infection (day −1), HAE were mock-treated or treated via the basolateral pole with 2 ng recombinant IFN-α2a or 20 ng recombinant IFN-λ1/IL-29, IFN-λ2/IL-28A, or IFN-λ3/IL-28B (PBL Assay Science) in 700 μl MucilAir culture medium. We assessed the functional neutralizing effect of anti–IFN-I antibodies, by incubating recombinant IFN-α2a, IFN-λ1/IL-29, IFN-λ2/IL-28A, or IFN-λ3/IL-28B (37°C, 1 h) with a 1% final dilution of inactivated (56°C, 30 min) patient plasma, containing or not containing anti–IFN-α antibodies, before addition to HAE. On the day after this IFN treatment, the apical poles of the HAE were gently washed twice with warm OptiMEM (Gibco, Thermo Fisher Scientific) and infected with 150 μl of a dilution of A/Lyon/969/2009 H1N1 virus in OptiMEM, at an MOI of 0.1, in the presence or absence of plasma. Basolateral treatment with recombinant IFN (with or without plasma) was repeated 1 h after infection in the same conditions as on day −1. Changes in TEER were measured with a dedicated Volt-Ohm meter (EVOM2, Epithelial Volt/Ohm Meter) and expressed in Ohm/cm^2^. At 54 h after infection, the apical poles of the HAE were washed with warm OptiMEM and collected in two tubes: one for TCID_50_ determination and the other for viral genome quantification by quantitative RT-PCR. HAE cells were harvested in RLT buffer (Qiagen), and total RNA was extracted with the RNeasy Mini Kit (Qiagen) for gene expression analyses.

### Transcriptomic analyses in reconstituted HAE

We hybridized 200 ng total RNA from HAE cells with a customized 96-gene panel, with counting on an nCounter FLEX platform according to the manufacturer’s instructions. Table S1 provides more information about the panel and the genes analyzed. Data processing and normalization were performed with nSolver analysis software (v4.0; NanoString Technologies), and the results are expressed as a fold-change induction relative to mock (untreated/uninfected) conditions. A heatmap of gene expression profiles from unsupervised hierarchical clustering (Euclidean distance matrix with Ward’s method) was generated with Genomics Suite 7 (Partek).

### ELISA

ELISAs were performed as previously described (Puel et al., 2022). In brief, 96-well ELISA plates (MaxiSorp; Thermo Fisher Scientific) were coated by incubation overnight at 4°C with 2 μg/ml rhIFN-α and rhIFN-ω (R&D Systems). Plates were then washed (PBS/0.005% Tween), blocked by incubation with the same buffer supplemented with 5% nonfat milk powder, washed, and incubated with 1:50 dilutions of plasma samples from the patients or controls for 2 h at room temperature (or with specific mAbs as positive controls). Each sample was tested once. Plates were thoroughly washed. HRP-conjugated Fc-specific IgG fractions from polyclonal goat antiserum against human IgG or IgA (Nordic Immunological Laboratories) were added to a final concentration of 2 μg/ml. Plates were incubated for 1 h at room temperature and washed. Substrate was added and OD was measured.

### Statistical analysis

OR and P values for the effect of auto-Abs neutralizing each type I IFN on critical influenza relative to healthy individuals from the general population, adjusted for age in four categories (<16, 16 to <50, 50 to <70, and ≥70 yr) and sex, were estimated by means of Firth’s bias-corrected logistic regression (Firth, 1993; Heinze and Schemper, 2002), as implemented in the logistf package of R software. We tested for an interaction between the effect of auto-Abs and age, by adding an age × auto-Abs interaction term to the logistic regression model, with age classified into two categories (<70 yr vs. ≥70 yr).

### Online supplemental material

Table S1 provides more information about the panel and the genes analyzed.

## Supplementary Material

Table S1 shows gene panel tested in the transcriptomic analyses in reconstituted HAE.
